# Using information communication technology to identify deficits in rural health care: a mixed-methods evaluation from Guatemala

**DOI:** 10.1080/16549716.2018.1455347

**Published:** 2018-04-17

**Authors:** Katharina Wahedi, Walter Flores, Claudia Beiersmann, Kayvan Bozorgmehr, Albrecht Jahn

**Affiliations:** a Institute of Public Health (IPH), Marsilius Arkaden, Heidelberg, Germany; b Centre for the study of Equity and Governance in Health Systems (CEGSS), Guatemala City, Guatemala, CA; c Department of General Practice and Health Services Research, University Hospital Heidelberg, Heidelberg, Germany

**Keywords:** Accountability, mHealth, eHealth, rural health care, Guatemala, mixed-methods, USHAHIDI

## Abstract

**Background**: In August 2014, the Centre for the Studies of Equity and Governance in Health Systems (CEGSS) in Guatemala launched an online platform, which facilitates complaints about health services via text messages. The aim is to collect, systemise and forward such complaints to relevant institutions, and to create a data pool on perceived deficits of health care in rural Guatemala.

**Objective**: To evaluate if the online platform is an accepted, user-friendly and efficient medium to engage citizens in the reporting of health care deficiencies in Guatemala.

**Methods**: The general study design of this research was a mixed-method approach including a quantitative analysis of complaints received and a qualitative exploration of the attitude of community leaders towards the platform.

**Results**: User statistics showed that a total of N = 228 messages were sent to the platform in the period August 2014–March 2015. The majority of complaints (n = 162, 71%) fell under the ‘lack of drugs, equipment or supplies’ category. The community leaders welcomed the platform, describing it as modern and progressive. Despite feedback mechanisms and methods to respond to complaints not yet being fully developed, many users showed a high intrinsic motivation to use the new tool. Others, however, were restrained by fear of personal consequences and distrust of the state’s judicial system. Access to mobile phones, reception, and phone credit or battery life did not pose major obstacles, but the producing and sending of correctly formatted messages was observed to be difficult.

**Conclusion**: The online platform paired with SMS technology appears to be a viable approach to collect citizens’ complaints in health care and connect citizens with relevant institutions. Further studies should be conducted to quantify follow-up activities and the impact on local health care provision.

## Background

Guatemala is the most population-dense and most economically powerful country in Central America. However, in 2013 more than half the population lived in poverty and 13.3% of the population was in extreme poverty [1]. Access to limited resources and infrastructure is especially difficult for the rural population. Although only 25% of the population live in the department of Guatemala City, the capital, most medical infrastructure and 80% of medical staff is concentrated there or in other urban and economically developed areas [2]. This results in severe shortages of medical provision in rural and underdeveloped areas, which strongly affects the indigenous population.

**Figure 1.**
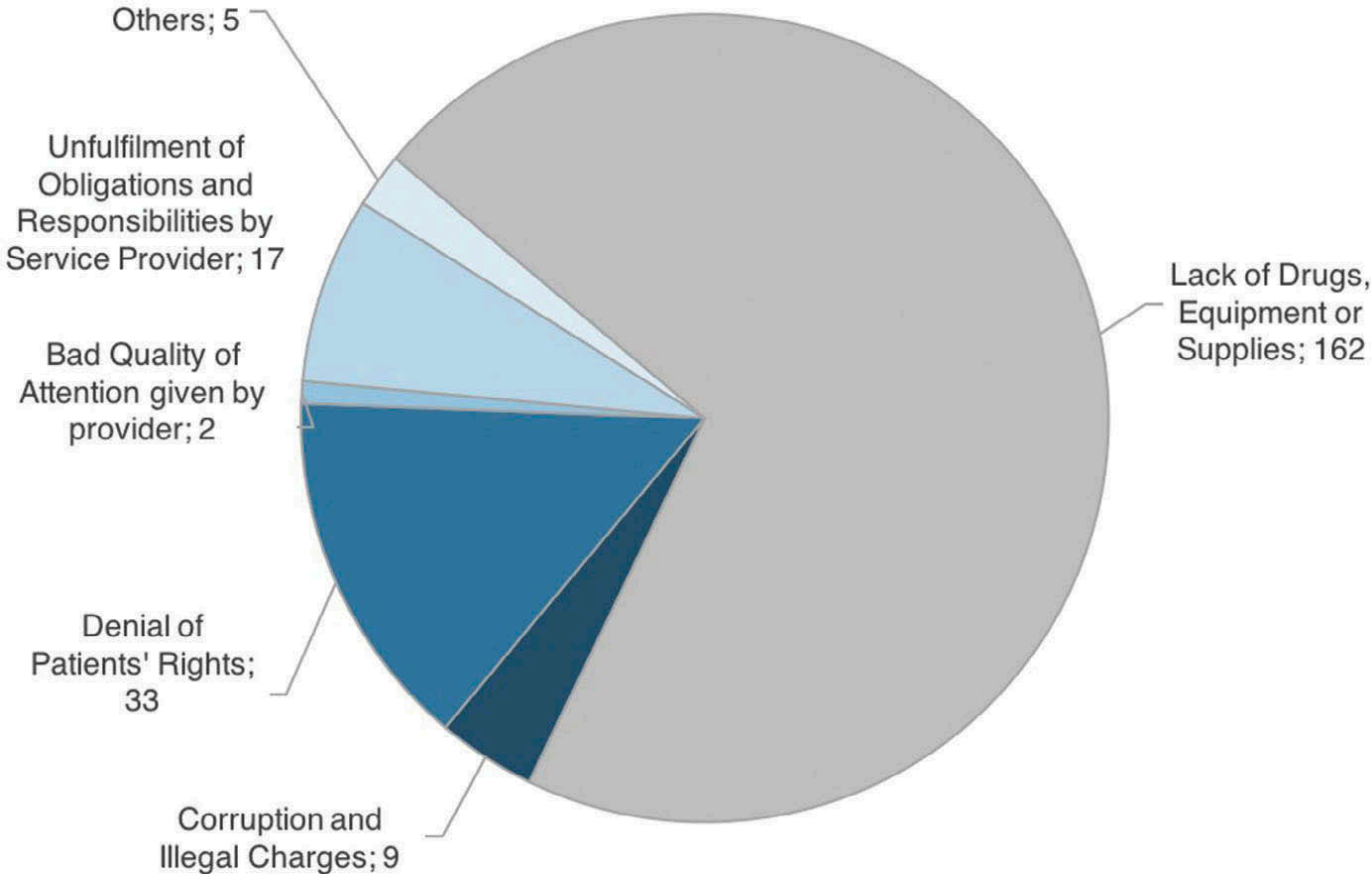
Complaints received between August 2014 and March 2015, sorted by complaint category.

Debates around accountability in the health sector intensified as the discussion of community participation and responsiveness of services developed. In rural settings in particular, the possibility for performance monitoring is limited due to a lack of resources, qualified personnel and infrastructure [3]. Common strategies by citizens to demand more accountability include publicly denouncing deficiencies in health care and calling on respective authorities to act more responsibly. As described by George [4], ‘although most situations conventionally do not favour marginalised groups who seek accountability, most authorities require a degree of public support and legitimacy to safeguard their status’. Vanhommerig and Karré [5] have described the way the internet has changed the role of citizens in public accountability by providing the possibility of cheap and instant communication. This has created new opportunities for citizens to interact with the government and create new spaces for political participation. They use the term *monitorial citizens* to describe the development of the civil society in its role as consumers of government services, interacting and demanding quality services rather than being mere recipients. Through the collection and creation of data sets, personal experiences can be aggregated and transformed into collective information and citizens can proactively hold the government accountable for structural problems and perceived deficiencies.

An example is the USHAHIDI platform (Ushahidi is the Swahili word for testimony), which was created by a Kenyan blogger, Ory Okollah, as a tool to collect and publish incidents of post-election violence in Kenya in 2008. In the beginning, she created software that collected information from different online sources such as Twitter and emails, linked it to a geographical location and subsequently presented it on a map. A non-profit software company, USHAHIDI, was founded based on this software, which then continued its development, as well as the development of other programs aimed to empower citizens to have a voice. They developed a feature called SMSlink, which allowed users to upload their experiences via SMS without having to access the online form [6]. The USHAHIDI platform was uploaded as an open access software and has since been used and adapted in many different countries and varying contexts [6]; for example, during the Congo conflict [7], road blockages in Washington, DC [8], the monitoring of elections in India [9], stock-outs of essential medicines in East Africa [10,11] (Banks, 2009) and the aftermath of the earthquake in Haiti [12].

In Guatemala, a system of *development councils* creates the designated space for social participation [13]. While well intentioned, there are several issues that prevent fair and effective use of this space, such as uncovered travel costs, and under-representation of and threats against civil representatives, as well as differences in power and education of users [14,15]

The Centre for the Study of Equity and Governance in Health Systems (CEGSS) has worked with the representatives of the communitarian development councils (the first of five successive levels: *consejo comunitario de desarrollo*, COCODE) in seven different states of Guatemala, engaging in rights- and participation-focused capacity building. Community members have since been engaged in various means of monitoring health services, including score cards and collecting ethnographic audiovisual data. In order to expand on these programmes, the CEGSS sought out a tool that could help systemise and organise incoming complaints, create publicly accessible statistics, and connect citizens with sources of potential help (e.g. ombudsmen of human rights [PDH], defenders of indigenous women). Hence, the USHAHIDI platform was adapted to collect and display citizen complaints about deficits in health care in Guatemala.

The overall objective of this research was to evaluate if the online platform is an accepted, user-friendly and efficient medium to engage citizens in the reporting of health care deficiencies in Guatemala. The specific objectives were: (1) the statistical description of the utilisation of the platform during the first phase of implementation; (2) the assessment of community leaders’ preference of complaint collection systems and experiences with the online platform; (3) the influence of political and social aspects on the motivation to file complaints; and (4) the technical feasibility of the online platform.

## Methods

### Study design

The general study design of this research was a mixed-methods approach including a quantitative analysis of incoming complaints and a qualitative exploration of the attitudes of community leaders towards the platform, and their personal experience using it.

### Study area

All interviews with community leaders were conducted with participants from the first round of workshops which had taken place in the seven departments of Alta Verapaz, Sololá, Quiché, Huehuetenango, Totonicapan, San Marcos and Quetzaltenango. These departments are predominantly rural with between 50% and 70% of the population living in rural areas. The departments of Alta Verapaz, Sololá and Quiché have the highest poverty rates found in Guatemala. Many of these departments have a high indigenous population and Mayan languages are more widely spoken than colonial Spanish.

### Quantitative data collection and analysis

Sending a complaint to the platform requires the user to choose a complaint code and a location code from a manual, which had been distributed and explained during introductory workshops between September and December 2015. The manual consists of a list of preselected complaints, which had been created based on previous reports and experience of the CEGSS, and validated through feedback discussions with community leaders. The list of codes entails 18 specific complaints in 6 categories, such as ‘A11’ or ‘D12’ (see Table 1). Location codes consist of a numeral code, specific for each health care centre. The final message requires only the combination of these two codes, e.g. ‘A11 170101’ for a lack of vaccines in the health centre of Lanquín.

**Table 1.** List of complaints received between August 2014 and March 2015, broken down for the specific complaints.

Complaint code	Category and complaints	Absolute	Percentage
	Lack of drugs, equipment or supplies	162	71,05
A11	Lack of vaccines	28	12,28
A12	Lack of drugs	123	53,95
A13	Lack of medical equipment and supplies	11	4,82
	Corruption and Illegal Charges	9	3,95
B11	Fee for medical service in Health Care Centre	3	1,31
B12	Fee for emergency transport	5	2,19
B13	Sale of drugs with stamp of MSPAS	1	0,43
	Denial of patients rights	33	14,47
C11	Due to lack of medical equipment or supplies	4	1,75
C12	Due to ethnic or idiomatic discrimination	13	5,7
C13	Due to missing health card	6	2,63
C14	Due to socio-economic, age or gender-based discrimination	3	1,31
C15	No informed consent	2	0,88
C16	Abuse of patients	5	2,19
	Bad quality of attention	2	0,88
D11	Dissatisfaction with the received treatment	1	0,43
D12	Lack of information given to the patient	1	0,43
	Failure of service provider to comply with responsibilities	17	7,45
E11	Health centre closed	15	6,58
E12	No assistance of the staff of the ‘extended coverage’ programme	1	0,43
E13	Infrastructure of health centre in bad conditions	1	0,43
F	Others	5	2,19
		228	100

The complaint data was obtained by downloading a data set of all complaints from August 2014 to March 2015 from the platform (accessible at www.vigilanciaysalud.com/plataforma.com). For each complaint it provided the date and time the complaint was received, type of complaint and location, and complaint ID (the ID code is unique for each complaint and resembles the number of complaints received by the platform since the launching of the website) (see Table 1). All complaint data was stored and managed with Microsoft Excel (Version 15), and used to create the statistics and design the diagrams.

### Qualitative data collection and analysis

Qualitative data was collected from interviews, observations in the field and relevant documents. Eighteen semi-structured interviews were conducted with community leaders as key informants. Interviewees were recruited through purposive sampling, including leaders who had previously collaborated with the CEGSS or participated in the initial introductory workshops. In order to incorporate different experiences, both leaders who had personally sent complaint messages to the platform and leaders who had not previously used the platform were recruited during meetings and workshops. An interview guide was created which included the following themes: general opinion and expectations towards the platform, reasons for using or not using the platform, usability and teaching workshops. Interviews were conducted by the first author as an external evaluator. All interviews were conducted and transcribed in Spanish, with the exemption of three interviews which were conducted in M’am and K’ekchi, with the help of bilingual community members.

Additional data was collected during a meeting of community leaders and politicians, two public complaint collection activities and four workshops. A document analysis of available protocols, and information material and informal conversations with involved staff members complemented the research.

Qualitative data analysis was performed based on a content analysis. Interview transcripts, documentation and notes from meetings and workshops as well as the additional data mentioned earlier were fed into MaxQDA (Version 12). A coding scheme was generated both inductively and based on the interview guidelines (deductively). After assigning codes to repeated ideas, these were grouped into themes and sub-themes as described in detail by Auerbach and Silverstein [16]. Based on these themes, theoretical constructs or categories were developed. Repeated ideas were coded and organised into themes and sub-themes and later summarised in theoretical constructs, such as the differentiation between the *method* of publicly collecting information on perceived deficits in health care and the *medium* through which this is achieved (i.e. the development of a technological approach connecting a web page with text messages).

### Ethics

This study was conducted in accordance with the Helsinki Declaration of 2013. Informed consent was gained by all participants of the study. The study protocol was submitted to the ethics commission in Heidelberg (S-030/2015) and approval was granted prior to collecting data.

## Results

### Summary of user statistics of the USHAHIDI platform in August 2014–March 2015

The total number of complaints received between August 2014 and March 2015 was N = 228. A first distinction has to be made between complaints concerning ‘a lack of drugs, vaccines, and medicines’ (category A) and all other complaints (categories B–F, see Table 1) due to their different legal implications. While the first resembles general misdemeanours of the state, such as not providing enough medicine or supplies, categories B–F include crimes against an individual person, which can be the base for filing criminal charges.

Figure 1 shows that the majority of complaints (n = 162, 71%) fall into this first category, while categories B–F make up the other 29% (n = 66). These remaining four categories are as follows: *Corruption and illegal charges* (n = 9; 3.94%), *Denial of patients’ rights* (n = 33; 14.47%), *Bad quality of attention given by service provider* (n = 2, 0.88%) and *Failure of service provider to comply with responsibilities* (n = 17; 7,46%).

Table 1 shows a more detailed distribution of the individual complaints within the categories: The two most common complaints are *Health centre closed* (n = 15) and *Denial of attention due ethnic or idiomatic discrimination* (n = 13). Furthermore Table 1 demonstrates that within the categories, some codes are evidently favoured.

### Leaders’ preference of complaint collection systems and experiences with the online platform

The majority of leaders displayed a positive attitude towards sending complaints via text messages to the online platform. Although learning how to use the platform and use the messaging codes was perceived to be a difficult process, most of the interviewed leaders gave preference to the platform over the previously used handwritten reports. When asked for the reasons behind their preference, most emphasis was put on the fast and instantaneous delivery of texts and lower costs as the expenses were limited to the costs of a text message rather than having to pay for travel costs and food during travel time:

Yes [I thought it was going to be complicated], but at the same time, I said, man, much faster, much more immediate. But it is hard, the format of the complete message. I see that it is very nice, because it goes faster. *Better than writing the complaints on the sheets?* Yes, a thousand times. *(Leader 1, St Cruz del Quiché)*

One leader reflected that the previously used written reports did not provide certainty as to whether the reports would arrive at the correct destination. However, the text messages provided a secure method of ensuring the complaint arrived directly at its desired destination. Other leaders described that the use of text messages had helped to reduce the sense of isolation in remote communities, since the delivery of text messages does not depend on infrastructure or public transportation.

On the other hand, some leaders said that whilst they appreciated the platform, they preferred to try to solve problems personally rather than sending reports to a different city. They also considered the complaining process as inefficient and taking too much time. Two leaders described that they preferred to continue using handwritten reports due to the complexity of the new complaint system and the technical challenges of composing correct messages.

Several leaders commented that they would like the platform to be extended to receive complaints beyond the context of health care, such as environmental concerns, social programmes or problems in the educational system.

### Technical challenges of the correct coding and sending of text messages

We found that that in order to send a complaint to the platform, the user has to have several different skills: (i) to find the adequate codes and (ii) to insert them into a message and successfully send the message to the right number.

(i) To find the adequate codes was not often seen as a reason for failing to send a message, but it made up a big part of the errors that occurred. During the workshops the first author noticed that the extraction of the correct codes posed a challenge for the leaders. The correct location code had to be found out of 200 possible codes and the appropriate complaint code had to be chosen according to the nature of the complaint. This posed an additional challenge as complex situations from real life had to be related to generalised and categorised codes, which required abstract thinking and a general understanding of the different codes. For example, relating personal experiences to codes such as ‘The doctor didn’t treat me’ or ‘negation of medical attention due to …’ not only requires abstract thinking, but also requires considering the motivation of the medical staff.

(ii) To put the identified codes into a message and send it successfully to the platform required a charged phone, reception and enough credit to send a message. Some leaders from very rural or particularly mountainous areas without adequate mobile coverage occasionally reported poor reception as a barrier to using the platform. More leaders experienced problems with the credit on their phones and many even reported they explicitly did not send messages due to insufficient credit. If all these prerequisites are met (credit, reception and correct identification of codes), the identified code has to be put in a text message. This caused the most difficulties amongst the leaders. In fact, five leaders said that before the workshop they had rarely or never used text messages as a means of regular communication.

Other leaders described how even though they used text messages on a regular basis for day-to-day communication, they faced problems sending messages to the platform. One woman stated:

‘Like I tell you, I know how to write messages, but it’s very different. I don’t know if you understand me, it’s very different’ *(Leader 3, Cobán)*.

Despite knowing how to send a regular text message, choosing and sending the code was often difficult for participants. Many leaders were using ‘frijolitos’, simple phones that do not have access to the internet and do not have a proper keyboard (QWERTY) but only 12 keys. As typing a code such as ‘B14’ requires changing between letters and numbers, writing out the code caused difficulties. Factors which were observed to facilitate learning included a young age, a newer phone and previous experience with text messages.

### Political and social aspects of leaders’ motivation to report incidents to the platform

Both political and social aspects were found to factor into leaders’ motivation to make use of the platform. Politically, many of the leaders had participated in local development councils for many years and showed a very high intrinsic motivation to report problems with health care provision in their communities. A generally shared aim is to improve the situation for themselves and their fellow citizens, as shown by the following quote:

Because [if] there’s no movement, nothing can get better. The people have to fight, they have to insist, complaining, complaining, to see changes. But if there are no complaints, it stays the same. … Maybe one person is not able to make a big movement by herself, but with all of the population you can make a grand movement. (*Leader 1, Cobán*)

Leaders were also motivated to collect and report problems because of their social standing in the communities they represent. Members of the community councils are known for their commitment to the community and hold respected positions. As part of another project initiated by the CEGSS, some leaders from the community councils personally collect information from the hospitals and had been given cards that identified them as ‘Defenders of Health’. They used these cards proudly and presented them whenever useful for them.

However, we also identified reasons that caused citizens not to report perceived injustices to their community leaders. Some community members feel a lack of trust towards the political system and a general fear of complaining and its consequences, as is shown by the following quote:

There are people who don’t want to complain. … Because maybe they will cause me problems, they say, where will you send the complaint, I don’t want to be in television, that’s what people say …, they are scared. It might turn into something political. *(Leader 4, Cobán)*

In addition, some leaders felt reluctant to publish complaints reported to them because the platform was a new system which had not yet become part of their routine. Whilst leaders stated their motivation to use the platform during and immediately after the workshops, we observed this positive response reduced over time. Other leaders stopped sending messages because the lack of confirmation that someone had received their message discouraged them. Some reported having been called by the CEGSS after having filed a report, but none described contact with human rights institutions or legal follow-up. Another factor observed to influence reporting and to explain the rapid decline of incoming messages was a lack of phone credit and/or an unwillingness to spend money on messages.

## Discussion

This study collected both quantitative and qualitative data on the use of the online platform by citizens from rural communities in Guatemala in order to evaluate whether an online platform with a text message function could be an accepted, efficient and usable medium for these communities.

### Key findings

In the period between August 2014 and March 2015, the first months after the first round of introductory workshops, a total of 228 received messages was registered by the platform. A high percentage of complaints addressed the lack of drugs, vaccines and resources, while complaints concerning abuse, discrimination or neglect of patients were also shared but not as numerous. Generally, the leaders appreciated and welcomed the technology, but with regard to usability, the sending of messages posed some challenges. The community leaders’ ability to successfully send messages varied greatly from easily learning how to send correct messages to failing to introduce the code correctly despite multiple supporting workshops.

### Implications of complaint statistics and leaders’ general attitude and experiences

While the content of these complaints and their implications will be discussed below, the mere number of complaints suggest that the platform met a need felt by the communities. Similar conclusions can be drawn from the interviews with community leaders. The majority of leaders expressed a preference for sending text messages over writing paper-based reports. They also favoured the lower costs and faster arrival, and expressed the preference to be called and report their experience verbally. However, over time, a decrease in messages received was noted. Reasons given by citizens which may have contributed to this development and may have caused a relevant under-reporting of experienced deficits include technical problems and difficulties in selecting the appropriate codes (elaborated below), issues with the cost of messages, minimal response to the complaints, and a decline in awareness of the platform due to a lack of reminders.

The cost of sending text messages posed a relevant access barrier to using the platform. Many leaders chose not to use the platform due to limited access to locations to buy phone credit or unwillingness to spend money on credit. In the future, attention should be focussed on finding strategies to solve this problem. Ideally this would be through establishing a phone contract for the phone connected to the web page, which allows the user of the platform to send messages free of charge, or developing reimbursement schemes for money spent on messages.

Action taken in response to the complaints is an essential factor for the success of any quality assurance concept. So far, reports from respective human rights non-governmental organisations (NGOs) and collective experience from the CEGSS showed that no cases had been given proper legal follow-up. There had, however, been successful administrative proceedings with authorities at municipal and provincial level. Consequences which resulted from these proceedings include: health staff which had been reported as abusive had to aplogise formally; staff were transferred to other provinces and official working hours were enforced [17]. The successful outcome of these particular cases shows the impact publicly accessible information can have on both informal and administrative procedures, as they depend – and in this also lies their limitation – on political will and receptiveness at the municipal level.

At the same time, many citizens expressed the wish to open the platform to other public services, such as education, cash transfers, agriculture or environmental pollution. This also demonstrates an appreciation of the online platform. Furthermore, the platform has since been requested by local health authorities to report problems with lack of supplies not properly addressed by the central-level government [18]

### Technological feasibility

A comprehensive discussion and evaluation of the online platform requires the differentiation of the two core aspects of the online platform: the *method* of publicly collecting information on perceived deficits in health care, and the *medium* through which this is achieved (i.e. the development of a technological approach connecting a web page with text messages).

With regard to the *medium*, the results of this study showed that in all communities there were enough leaders who owned a phone and that all interviewed leaders had sufficient access to electricity; none reported having experienced problems with charging batteries. Even though some of the leaders from the most remote areas reported an occasional lack of reception, it did not present insurmountable barriers for them to use the platform, as they were either temporary or geographically limited. These findings suggest that the technical prerequisites were met and that the reporting of complaints of deficits in health care via mobile phones is a viable approach for rural communities in Guatemala.

The spread of the Internet has decreased the costs of communication and increased the accessibility of information and the speed of data collection. Many poor and rural areas could benefit immensely from this, but access to the internet is still often limited to metropolitan areas. World Bank statistics show that only 23% of the Guatemalan population has access to the internet; however, an average of 107 phones are registered per 100 people in Guatemala [19]. This demonstrates that mobile phones with internet access are not very common. The pairing of the USHUAHIDI platform together with the SMSSync program makes use of text messages as a medium to enable citizens to upload information to the Iinternet without the necessity of having access to the Iinternet.

The ethnic and idiomatic diversity of Guatemala is an important characteristic of the country and a relevant factor to the quality of health service received. Those affected most by deficiencies of the public health care are the rural and indigenous population. They report fewer doctor visits and longer travel times to reach the service, and are half as likely to attend preventive consultations than the non-indigenous population [20]. A relevant portion of the population is not able to communicate adequately in Spanish, which hinders communication outside their idiomatic group [21]. In this context, the technological approach of the platform was observed to be incredibly helpful. The complaint is passed on through a code that community leaders can find using manuals in their native languages. Subsequently, the complaint can be displayed on a map with a Spanish description. In the second phase, the affected person will be called by a lawyer – either from the CEGSS or an NGO – who will request further information. This person may be chosen based on the language the affected person prefers. Both mechanisms use the technological approach to minimise language barriers and maximise effective communication for non-Spanish speakers.

### Political and social implications

Following the differentiation of two core aspects medium (Italian letter) and method (Italian letter), the method applied by complaining through the online platform is to collect information on deficits in health care in order to demand accountability from responsible authorities or structures. The results show that, in principle, community members appreciated the importance of the procedures and potential outcomes of legal proceedings to pursue government accountability for human rights violations. However, this is also in conflict with a deep-seated distrust of the legal system, as citizens frequently cited fear of reprisal as a reason not to pursue individual complaints through the judiciary system. In Guatemala, impunity and lawlessness are chronic problems of the judicial system. High levels of violence are met with a lack of financial resources, corruption and infiltration by illegal and clandestine security organisations [22]). A recent article published in *Prensa Libre*, an established Guatemalan newspaper, describes a conviction rate of 2.96% for all cases of corruption in 2015 [23]. Similarly, a report by Freedom House describes the general conviction rate as less than 2% or impunity rates of around 98% [24]. Further access barriers to fair processes are social exclusion due to language barriers or geographic isolation [25]; a high economic burden due to court fees, travel costs, lost work days and the need to contract a lawyer [26]; and a historic hesitancy about engaging with state institutions. This context raises a question on the rationale of improving the health care provision by demanding legal accountability from state institutions.

In addition, the results show that a high percentage of complaints addressed the lack of drugs, vaccines and resources, while complaints concerning abuse, discrimination or neglect of patients were also reported but not as numerous. This impacts the range of action which could be taken to address the complaints. A lawsuit can only be filed on individual cases when a crime has been committed against a patient, or a patient’s rights have been violated. In consequence, all complaints addressing a general lack of drugs, vaccines and resources did not receive individual follow-up, but instead were simply documented. This may have an effect in itself, as it can be of use for political advocacy and publicity within a civil society movement. However, the results for the individual will not be as prompt and direct compared to attending court and pressing charges. Regardless, short-term change is important to demonstrate the authorities’ responsiveness and willingness to address problems and to maintain the motivation of communities to continue their engagement with authorities.

### Strengths and limitations of the study and study design

The study design and point of time of this study were not laid out to quantify follow-up to the complaints, and perceived changes in health care in response to the complaints. Processes of data derivation and response protocols with human rights institutions were not yet fully developed for conclusions on responsiveness and effects on health care. The statistics obtained through the complaints registered by the platform do not allow for general assumptions about perceived deficits in health care in rural Guatemala, as they only resemble the experiences made by selected leaders in selected communities in the respective departments. However, they demonstrate the strong need and desire of citizens for public data collection. The qualitative section of this study allows for a deeper understanding of citizens’ underlying motivation to send or not to send complaints and allowed for modification of the processes tailored to citizens’ needs.

## Conclusion

Our results indicate that the platform met a relevant need in the communities studied and that its technology was welcomed for its advantages and innovation. Usability and costs of text messages posed problems for some, and strategies to resolve these issues have to be prioritised. As a large percentage of the complaints received concerned systemic deficits rather than violations of individual patients’ rights, responses have to focus on achieving accountability through both direct and legal follow-up to individual complaints, but also general, administrative consequences. Likewise, they should focus on the collection of information to further collaborate and overcome underlying deficits of the national health system. Further studies should focus on analysing the follow-up processes and evaluating perceived change in the provision of health care in Guatemala.

## Paper context

The USHADHI platform had successfully been applied in multiple contexts, within and outside the health care context. In 2013–2014, the centre for the studies of equity and governance in health systems with the support of the University of Parsons, New York worked on launching the USHAHIDI platform with the aim to monitor health care in Guatemala. The research presented in this study formed an integral part of the evaluation process.
